# The effect of waiting on aggressive tendencies toward emergency department staff: Providing information can help but may also backfire

**DOI:** 10.1371/journal.pone.0227729

**Published:** 2020-01-29

**Authors:** Dorit Efrat-Treister, Hadar Moriah, Anat Rafaeli

**Affiliations:** 1 Department of Management, Ben-Gurion University of the Negev, Beer Sheva, Israel; 2 Faculty of Industrial Engineering and Management, Technion-Israel Institute of Technology, Haifa, Israel; University of Milan, ITALY

## Abstract

**Introduction:**

Waiting is inevitable for customers of service organizations, yet having to wait can trigger aggression by care receivers toward hospital staff. We explore the effect of waiting times on care receivers’ sense of procedural justice and aggressive tendencies, and show the attenuating effects of providing explanatory information.

**Methods:**

Data were collected using survey responses in two studies, both conducted in the waiting area of a large hospital emergency department. Study 1 (n = 328) was a quasi-experiment involving an intervention in which care receivers were provided with information about wait times. Study 1 included three phases: (1) pre-test (week 1, n = 98), in which no information was provided; (2) information condition (weeks 2 & 3, n = 155), in which information was provided through large signs and pamphlets; and (3) post-test (week 4, n = 75), in which no information was provided. Study 2 (n = 99) was conducted a year later and involved the same information provision as the intervention stage of Study 1.

**Results:**

The longer the wait duration, the lower care receivers’ procedural justice perceptions and the greater their aggressive tendencies. Information provision moderated the association, such that receiving information reduced aggressive tendencies during shorter waits but increased aggressive tendencies during longer waits. We show these effects in two separate data collections, conducted one year apart.

**Conclusion:**

Competing theories predict that explanatory information should variously reduce and increase aggressive tendencies among people waiting in a queue. Our findings resolve this contradiction by identifying boundaries for the effectiveness of providing information in reducing aggression. We show that providing information is likely to reduce aggression until such point as the wait duration becomes longer than expected based on the information provided.

## Introduction

“Knowledge is power. Information is liberating.”

- Kofi Annan

Queueing to obtain a desired service is an inevitable aspect of modern societies [1]. However, while queueing is necessary to obtain service, it is unpleasant and embodies the frustration of delaying gratification. As such, it may fuel aggression among those waiting to receive the service [2, 3]. Aggression, in turn, elicits feelings of anger and frustration in the target of the aggression [4], and thus reduces well-being [5]. Most service employees encounter customer aggression frequently [6, 7]. Hence, examining means of reducing aggression among service receivers is important.

The present study examines the effect of waiting on aggression among a certain group of customers–namely, health care receivers (patients and escorts) waiting to receive service from hospital staff. Limited research has examined aggression of customers against employees in general [8], and aggression of care receivers against hospital staff in particular. Even more limited is research into means of buffering such aggression [9]. In the current study, we join scholars who suggest that the availability of information can influence people’s reactions to waiting (e.g., [10, 11]), and examine whether information provision can provoke or attenuate aggressive tendencies among care receivers waiting for service. In so doing, we build on previous research on aggression in hospitals (e.g., [12]) and organizational justice theory [13, 14, 15]. However, unlike these classic works, we show that in the case of waiting, providing information has a complex influence that can either attenuate or increase incidents of aggression. The present study reports on a field experiment in a hospital Emergency Department (ED) in which we examined the moderating role of information in the relationship between time waited and aggressive tendencies. Building on previous research showing that aggressive tendencies predict actual aggressive behavior [16], we used aggressive tendencies as a proxy for aggression in our dependent variable.

Information about waiting is unique in that it refers to events that are yet to occur (i.e., information about how long one will have to wait is given before one actually receives service). Yet available studies have primarily examined the effects of information about events that have already occurred. For example, studies have examined effects of information about past adverse organizational procedures on trustworthiness [17], and effects of information about past market performance on financial decisions [18].

In contrast to such “post-hoc” information, information about events that are yet to occur is inherently uncertain, and its accuracy is unclear at the time of delivery [19]. As such, providing information about future events can be considered a means of framing expectations [20, 21]. We examine information about waiting times as a case of information about future events, and presume that such information serves mainly to establish expectations about how long one will have to wait. We predict that such expectations influence people’s behavior, with different effects depending on whether or not the expectations are met.

The difference between information about events that have occurred and information about events that are yet to occur can help make sense of mixed findings in previous research regarding the positive versus negative effects of providing people with information about organizational procedures. On the one hand, information about events that have already occurred enhanced people’s understanding of the reasons for a wait, and thereby improved their sense of justice about the situation [22–27]. In contrast, information about future events can evoke negative reactions, because it creates expectations that may or may not be met [28, 29]. Failure to meet those expectations can be construed as a sign that the organization is at fault [11], leading to negative reactions. In this vein, Mandelbaum and Zeltyn [30] found that customers waiting in a telephone queue were more likely to hang up after hearing information about the expected wait; and among those who stayed on the line, negative reactions ensued if the customer’s expectations were not met [31–33]. For example, a half-hour wait may be more annoying for someone previously informed that their wait would be twenty minutes compared with someone given no information about the likely wait time at all.

Our study makes three contributions to the existing literature. First, it documents the effects of pre-emptive information–namely, information provided before people endure an aversive situation–in the context of waiting. Second, it untangles the positive and negative effects of providing information, establishing boundaries for the effectiveness of providing information about waiting. Third, it demonstrates the risks of providing information that can generate false expectations.

## Theoretical background and hypotheses

### Waiting duration and aggressive tendencies

Waiting is frustrating because it obstructs goal attainment [34–37]. As such, having to wait can be seen as a source of stress. Stress, and the frustration that accompanies it, need to be managed through continuous self-control and regulation of emotions–abilities that are depleted over time [38]. This depletion of self-control and regulatory capacities increases the risk that frustration will boil over into aggression [39, 40].

We build on previous research [41, 42, 43] in defining aggression as acts carried out with the intention of causing harm to an individual or an organization. Our measured variable is aggressive tendencies, defined as people’s tendency to yell, curse, insult or ignore staff, damage equipment, or interfere with work processes [44]. Such tendencies, variously referred to as uncivil [45], deviant [46], or retaliatory behaviors [47], are likely to escalate into more severe aggressive behaviors [16]. They are also alarmingly common [48] and are likely to be predicted by situational triggers [49]. More extreme acts of aggression rarely occur in response to triggers such as waiting [50], and are, therefore, not included in our analyses.

Waiting can be seen as a hindrance stressor, defined as “demands that are perceived as hindering progress toward personal accomplishments or goal attainment” [51, 52]. Hindrance stressors are known to elicit frustration and, in turn, aggression [53]. In queues, the basic dynamic that drives the development of such a hindrance stressor is the sense of time being lost or wasted (rather than being used to accomplish a goal). It follows that the longer one must wait, the greater the hindrance; the greater the hindrance, the greater the stress; and the greater the stress, the greater the risk of aggression. Accordingly, our first hypothesis is as follows:

Hypothesis 1: Wait duration predicts people’s aggressive tendencies while waiting.

### Waiting duration and procedural justice

Wait duration is an objective aspect of waiting–but how people experience time is subjective. Another aspect of waiting with objective and subjective aspects is the rules by which the queue operates, and specifically their effect on queuers’ sense of procedural justice. Queues that follow a First-Come-First-Served (FCFS) principle are generally perceived as fair [54, 55], while deviations from the FCFS principle often violate procedural justice and create a sense of unfairness [10, 36]. Procedural justice refers to perceived fairness in the decision rules by which resources are allocated [56–58]. Hence, the policy that determines the next person to receive service establishes the procedural justice of the queue. Other forms of justice can also play out in waiting, including distributive justice (perceived fairness in the allocation of goods) and interactional justice (the degree to which people affected by decisions feel they have been treated with dignity and respect) [56]. But we suggest that procedural justice is the form of justice most relevant to the study of queues, where the good being allocated (service provision) is identical for everyone, but can only be supplied to one person (or a few people) at a time.

The context of the present research–waiting for service in a hospital emergency department–is especially challenging, because waits in this context can be long, and anxiety is high to begin with. Anxiety increases the sense of frustration and unfairness that tend to accompany long waits, leading to more negative responses as the duration of the wait increases [10]. This effect is exacerbated by people's general tendency to focus on their own needs rather than on the needs of others [59]. As waiting durations increase, more opportunities accrue for people to attribute their wait to violations of procedural justice in the queue. Thus, our second hypothesis connects wait duration to perceptions of procedural justice:

Hypothesis 2: Wait duration is negatively correlated with perceived procedural justice, such that the longer the wait duration the less the queue is perceived as procedurally just.

### Procedural justice and aggressive tendencies

Procedural justice is the type of justice most strongly related to counterproductive work behaviors [56]. Similarly, the negative reactions evoked by perceived procedural injustice can trigger aggressive tendencies [e.g., 14, 44, 60]. Meta-analyses of studies on justice [13] and on aggression [61] report moderate to strong negative correlations between perceptions of justice and aggression. In the broader context of social exchange theory [62, 63], aggression can be viewed as a form of repayment for perceived injustice [61, 64]. Of the different types of perceived justice, procedural justice is the best predictor of aggressive tendencies, more than distributive and interactional justice [63]. Hence our third hypothesis:

Hypothesis 3: Perceived procedural justice is negatively related to aggressive tendencies.

Hypotheses 1–3 connect wait duration and perceived procedural justice to aggressive tendencies when queueing. H1 suggests that aggressive tendencies may arise directly in response to the stress of queueing, while H2 and H3 suggest an indirect effect of wait duration on aggressive tendencies, through perceived procedural injustice. Our fourth hypothesis makes this mediated relationship explicit:

Hypothesis 4: Perceived procedural justice mediates the relationship between wait duration and aggression.

### The complex effects of explanatory information on aggressive tendencies

A final and central part of our theory regards the complex effects of information on the relationship between wait duration and aggression. We propose two related dynamics. First, we argue that providing information about the expected duration of the wait creates expectations in queuers [65], and that these expectations affect the relationship between the wait duration and aggressive tendencies. More precisely, we propose that queuers maintain their equilibrium as long as they believe their expectations about the length of their wait will be met. As the wait approaches its expected end point with no sign of actually ending, people’s anxiety and dissatisfaction build and may find an outlet in aggression [66–68]. Second, and relatedly, we suggest that the accuracy of the expectations created by provided information is central to whether information provision reduces or increases aggression. Specifically, information that creates accurate expectations has a beneficial effect, easing the wait for queuers and reducing aggression. Information that creates false expectations has a deleterious effect, making the wait more frustrating and increasing aggression.

To put it differently, when no information is provided, no expectations are set. In this case, the relationship between the wait duration and aggressive reactions should be stable, and high, from the beginning of the wait. In contrast, when information is provided, we expect the relationship between wait duration and aggressive tendencies to be steep, with an upward slope. In these cases, the initial level of aggressive tendencies should be significantly lower than when no information is provided. However, as the wait time lengthens and approaches the target created by the information, the slope reflecting the relationship between wait duration and aggressive tendencies becomes ever steeper. At some point, at which the time already waited meets or exceeds the expected total wait duration with no end in sight, aggressive tendencies will be equal to or even higher than if no information had been provided. This is the point where we posit that providing information backfires. Thus, we predict:

Hypothesis 5: Presented information and wait duration interact to influence aggressive tendencies, such that aggressive tendencies rise as the target wait time established by the information approaches. Once that target has passed, aggressive tendencies rise to a level higher than the baseline of no information.

**Fig 1:**
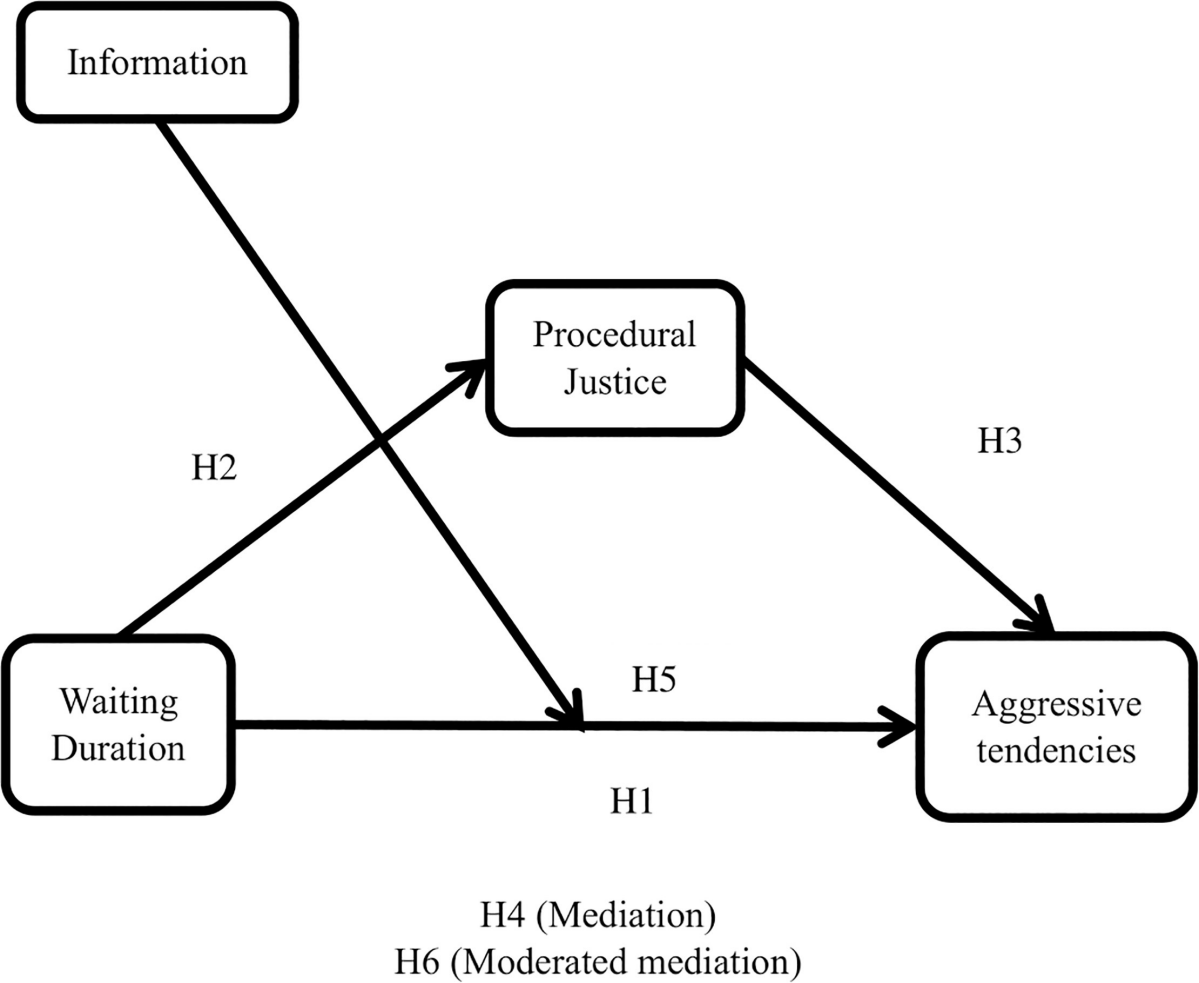
Research model and hypotheses.

Taken together, our five hypotheses predict a moderated-mediation model, as depicted in Fig 1. Our last hypothesis integrates Hypotheses 1–5:

Hypothesis 6: Perceived procedural justice mediates the relationship between wait duration and aggressive tendencies. Providing information initially reduces aggressive tendencies but then leads to a stronger positive relationship between wait duration and aggression.

We tested our hypotheses in a field stimulation study in the ED of a large public hospital, a setting that frequently involves extended waiting times. In Study 1, we tested our predictions by comparing people's aggressive reactions in two conditions: a no-information condition, and an information condition in which information pointing to likely wait durations was provided. We also measured perceived procedural justice as a mediating mechanism. Following the results of this study, which showed reduced aggression in the information condition, the distribution of information in the ED became part of the hospital routine. Then, a year later, we again collected data from the same hospital (Study 2). The results of that study were similar to those of Study 1, pointing to the enduring nature of the described effects.

## Study 1

We conducted a between-subjects field experiment [69]‏, following the guidelines of Hagger and Luszczynska [70] on the implementation of interventions in health contexts. In a randomized controlled study design, we presented information about hospital procedures to care receivers in the waiting area (queue) of the ED in a large public hospital. We then collected survey responses from a sample of those individuals to assess their perceptions of procedural justice and their aggressive tendencies, along with control variables (see below). As a control condition for comparison, the same data were collected from care receivers in the waiting area during the weeks prior to and following the experimental intervention.

### Methods

#### Ethics approval

This study was approved by the hospital ethics committee: 'HELSINKI committee Rambam Medical Center; Approval number: 0216-09-RMB, and by the Technion- Israel’s Institute of Technology IRB ethics approval; Approval number: 2019–010. Written consent was obtained. No minors were included in this study.

#### Participants and procedure

We collected data in three phases: (1) pre-test (week 1, n = 98), in which no information was provided; (2) information condition (weeks 2 and 3, n = 155), in which information was provided through large signs and pamphlets; and (3) post-test (week 4, n = 75), in which no information was provided. To control for potential changes unrelated to the information condition, the post-test condition was identical to the pre-test condition in all respects. Participants were care receivers waiting in the queue of the hospital's ED (n = 328; M_age_ = 36.48 years, SD_age_ = 16.02 years; 52.1% males; 49%). They were approached and agreed to respond to a short survey in return for a free beverage from the hospital’s coffee shop. Research assistants visited the ED on random days and hours to collect the survey data. Ninety-six percent of the people approached agreed to respond to the survey.

#### Presented information

We decided what information to present based on preliminary observations in the ED, interviews with ED staff, and a sample of care receivers. The chosen information, which was not previously available to people waiting in the queue, was presented in the form of a flow chart describing the various stages encountered by a typical visitor to the ED, including expected wait durations for the various stages of the visit (e.g., two hours for blood test results; five hours for the total average stay in the non-ambulatory ED ward, etc.; see S1 Appendix). The durations of the various stages of the visit were calculated based on interviews with the ED staff and hospital archival data regarding average wait times for each stage. In the information condition, the information was shown simultaneously on large signs in the ED waiting area and in pamphlets handed out by the ED receptionist when new arrivals checked in.

### Measures

**Manipulation check.** Following Colquitt [71], we asked participants to respond to items that assessed informational justice (namely, the appropriateness, honesty, and adequacy of any information provided), and items assessing clarity of the specific information provided in the intervention.

**Informational justice** was measured using a three-item measure adapted from Li et al. [72]: “The information I received was given in… a candid and direct fashion / an honest fashion / an explicit fashion” (Cronbach's α = 0.93; Cronbach's alpha represents the reliability estimate of a psychometric test). Responses were given on a 7-point Likert scale, with 1 = "I agree to a very small extent" and 7 = "I agree to a very high extent."

**Informational clarity** was measured using a four-item measure based on Brockner et al. [73]: “The information in the poster and pamphlet is generally clear and understandable"; “The information about the functioning of the ED is clear”; “The information about the ten things that I need to know in the ED is clear”; “The hospital map is clear” (Cronbach's α = 0.85). Responses were given on a 7-point Likert scale, with 1 = "I agree to a very small extent" and 7 = "I agree to a very high extent.” The informational clarity items were presented only to people in the information condition.

**Wait duration** was measured as time elapsed since the participant's arrival in the ED at the point where he/she responded to the survey. It was based on two questions: “When did you arrive at the ED?” (arrival time) and “What time is it now?” (current time). The duration of the wait was calculated as the difference between the arrival time and current time.

**Procedural justice** was measured using a seven-item scale adapted from Li et al. [72] and Gilliland et al. [74]: “The ED functions in a proper manner”; “There is method in the way the ED functions”; “I understand the order in which people are served”; “I understand why I am waiting”; “The order in which people are served is determined justly”; “The order in which people are served is determined fairly”; “The duration of the wait in the ED is determined in a just manner” (Cronbach's α = 0.92). Responses were given on a 7-point Likert scale, with 1 = "I agree to a very small extent" and 7 = "I agree to a very high extent.”

**Aggressive tendencies.** We could not measure actual aggression, because people who exercise aggression are frequently removed from the ED, and in any case are not available to respond to surveys. As a proxy, we measured people’s tendency to act with aggression, building on previous research which showed that aggressive tendencies are valid predictors of actual aggressive behavior (e.g., [16]).

We developed a self-report scale of aggressive tendencies by adapting Glomb [63] to the ED context, as recommended by Hofmann et al. [75]. We started with a list of 44 aggressive acts, based on observations at the ED, interviews with ED staff, and a literature review. In a pilot study, undergraduate students (n = 43; M_age_ = 25.23 years; 63% female) rated the level of aggressiveness, relevance to the hospital setting, and clarity of these 44 items, using a 7-point Likert scale (1 = "to a very small extent"). The ratings of the full set of items yielded M_aggressiveness_ = 4.84 (SD = 1.45); M_relevance_ = 4.39 (SD = 0.88); and M_clarity_ = 6.49 (SD = 0.28). We then dropped items that were rated below the mean score for relevance and items rated below -1SD for clarity. This yielded a seven-item measure: “I would like to … use an aggressive tone of voice toward a staff member / yell / barge into the office (i.e., go in without being called) / curse / bang on a table / slam a door / interrupt a staff member” (Cronbach's α = 0.73).

**Control variables.** Following Carlson and Wu [76], we controlled for several variables for which there is theoretical basis to predict influence on the dependent variables (procedural justice and aggressive tendencies). Control variables included number of medical interactions already received; time of day; and demographic variables. There were no significant differences in the control variables between the care receivers who received information and those who did not receive information, confirming that the assignment into the information vs. control condition was random (see S2 Appendix).

**Number of medical interactions (NMI).** The NMI was defined as the number of times a participant reported having been served by medical personnel and/or undergone medical tests in the course of their current visit to the ED. This serves as a proxy for severity of the patient’s medical condition and for familiarity with ED procedures, which might affect wait duration expectations, perceived procedural justice, and in turn, aggression.

**Time of day.** Time of day is known to influence positive affectivity [77], and aggression [78]. Specifically, morning and night are known to have lower levels of positive affectivity, than mid-day. Following Spector et al. [79], who argue that it is important not to control for affectivity in research concerning job stressors (such as waiting), we controlled for time of day as a proxy for affectivity.

**Demographic variables.** Participants were asked to indicate their gender, age, years of education, and number of visits to the ED in the past three years.

### Results

#### Manipulation check

Analysis of the information clarity measure confirmed that the information presented as part of the intervention was clear (M = 6.18, SD = 0.83). An ANOVA showed that perceived informational justice was significantly affected by the study condition (F _**(1,326)**_ = 7.76, p < 0.01), with significantly higher informational justice in the information condition (M = 4.55, SD = 2.13) than in the pre-test (M = 3.51, SD = 1.96, p < 0.01) and post-test (M = 3.32, SD = 2.20, p < 0.01) conditions. There was no difference in informational justice between the pre- and post-test conditions (p = 0.54), confirming that the manipulation was successful in providing information that was otherwise unavailable to the participants. As there were no significant differences between the pre-test and post-test conditions in any of the study variables (waiting duration, procedural justice, aggressive tendencies), these conditions were combined and we refer to them through most of our analyses as the no-information condition.

#### Wait duration, justice perceptions, and aggressive tendencies

Table 1 summarizes the means, standard deviations, and inter-correlations between all the study variables and control variables. Hypotheses 1–3 were tested using a regression analysis of the full dataset, including the three study conditions, and controlling for the effects of time of day, NMI, and demographic variables. The analyses confirmed a positive relationship between wait duration and aggressive tendencies (β = 0.22, p < 0.001), supporting Hypothesis 1; a negative relationship between wait duration and procedural justice (β = -0.29, p < 0.001), supporting Hypothesis 2; and a negative relationship between procedural justice and aggressive tendencies (β = -0.14, p < 0.05), supporting Hypothesis 3. None of the control variables were found to influence either aggressive tendencies or procedural justice (n.s).

**Table 1:** Means, standard deviations, and correlations among study variables.

	Mean	SD	1	2	3	4	5	6	7
**1. Wait duration**	2.90	2.22	-						
**2. Procedural justice**	3.88	1.68	-.136*	-					
**3. Aggressive tendencies**	1.97	1.86	.124*	-.259**	-				
**4. Age**	36.54	16.02	.09	.102†	-.119*	-			
**5. Gender**	.46	.50	.08	-.07	.06	.04	-		
**6. Years of Education**	12.57	4.24	.07	-.07	.01	.00	.09	-	
**7. NMI**	2.68	2.24	.520**	.185**	-.01	.08	-.02	.11	-
**8. Time of day**	3.67	1.27	.235**	.00	-.03	.148*	-.03	.198**	.257**

N = 312, Sig. (2-tailed)

†p < .1

*p < .05

**p < .01

*** p < .001; Wait duration is measured in hours, such that 3.67 stands for 3:42 pm.

The mediating role of procedural justice in the relationship between wait duration and aggressive tendencies was tested using Hayes’s [80] model 4, bootstrapped sample = 5000. (Bootstrapping is a resampling technique used to obtain estimates of summary statistics. It allows assigning measures of accuracy to sample estimates. The bootstrapping of 5,000 was chosen based on [80]). As predicted, wait duration had a negative influence on procedural justice (β = -0.24, p < 0.001, CI = -0.35, -0.14) and a positive influence on aggressive tendencies (β = 0.15, p < 0.01, CI = 0.04, 0.26). The indirect effect of wait duration on aggressive tendencies via procedural justice was significant (indirect effect = 0.04, CI = 0.01, 0.08; R^2^ = 0.10, p < 0.001, boot = 5000). These results support Hypothesis 4 (see Table 2).

**Table 2:** Simple mediation (Hayes [80], model 4) and moderated mediation (Hayes [80], model 5) predicting aggressive tendencies.

			Model 4	Model 5
		Procedural justice	Aggressive tendencies	Aggressive tendencies
		β(SE)	β(SE)	β(SE)
**Constant**	** **	4.04*** (.45)	3.59*** (.55)	3.85*** (.56)
**Waiting duration**	** **	-.23***(.05)	.01† (.06)	.02(.07)
**Age**	** **	.01† (.01)	-.01* (.01)	-.01* (.01)
**Gender**	** **	-.12 (.19)	.09 (.21)	.11 (.21)
**Education**	** **	-.03 (.03)	-.03 (.03)	-.04 (.03)
**NMI**	** **	.26***(.05)	-.02 (.06)	-.04 (.06)
**Time of day**	** **	-.03(.08)	-.03(.09)	-.01(.09)
**Procedural justice**	** **		-.24*** (.06)	-.23*** (.06)
**Information**	** **			-.73* (.36)
**Procedural justice x Information**	** **			.19* (.1)
**Indirect effect of Wait on Aggressive tendencies via procedural justice**	** **		.06*(.02)	.05*(.02)
**Conditional direct effect of Wait on Aggressive tendencies**	**No information**			.02(.07)
	**Information**			.21**(.08)
**R**^**2**^		.11***	.09***	.11***
**ΔR**^**2**^	** **			.01*

†p < .1

*p < .05

**p < .01

*** p < .001

#### The moderating influence of information

**Fig 2:**
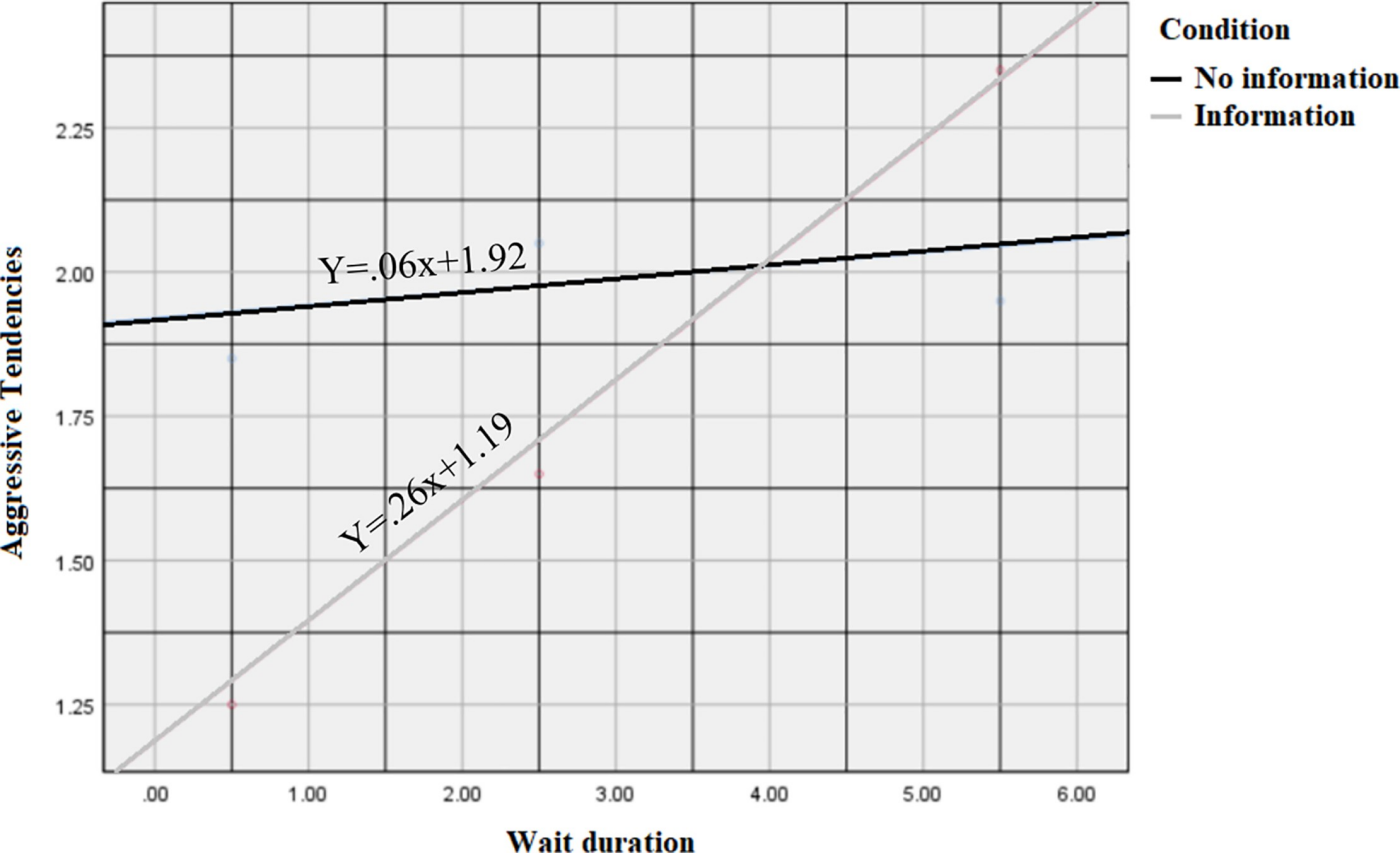
Simple slopes illustrating the influence of wait duration on aggressive tendencies, with and without explanatory information.

We found a significant interaction between presented information and wait duration in their effect on aggressive tendencies (β interaction = 0.20, p < 0.05, CI = 0.02, 0.39), supporting Hypothesis 5. As evident in Fig 2, when no information was distributed, there was no influence of wait duration on aggressive tendencies. However, when wait duration approached expectations (at 4 hours, when the expectation was 5 hours), the provision of information backfired, and care receivers showed more aggressive tendencies than when no information was provided.

**Fig 3:**
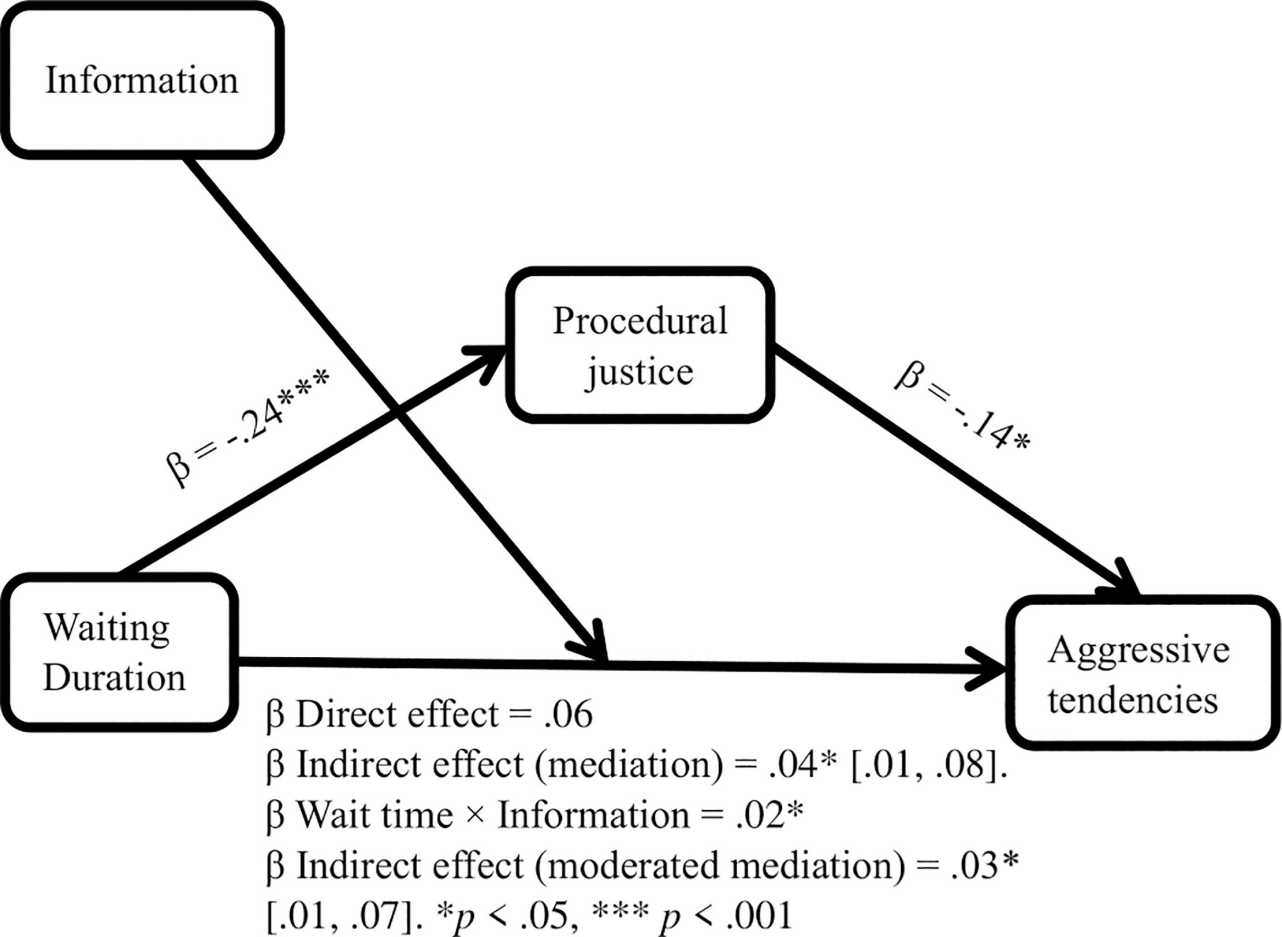
Summary of the results of research model.

Next, Hypothesis 6 was tested using Hayes’s [80] model 5, bootstrapped sample = 5000. As predicted, providing information moderated the direct effect of wait duration on aggressive tendencies (β interaction = 0.20, p < 0.05, CI = 0.02, 0.39). Wait duration increased aggressive tendencies in the information condition (slope = 0.26, p < 0.001, CI = 0.11, 0.41), but not in the no-information condition (slope = 0.06, p = 0.42). The slope in the information condition differed significantly from that in the no-information condition (p < 0.01). In addition, procedural justice mediated the indirect effect of wait duration on aggressive tendencies (β = 0.03, CI = 0.01, 0.07; R^2^ = 0.10, p < 0.0001). Thus, providing explanatory information to people waiting for service in the hospital ED was associated with a significantly stronger relationship between wait duration and aggression, supporting Hypothesis 6 (see Table 2, Figs 2 and 3).

## Discussion

The results of Study 1 fully support our hypothesis that the longer people must wait to be served, the less they perceive organizational procedures as just, and–at least partly in consequence–the more aggressive they tend to be. In addition, providing explanatory information about the reasons for and likely extent of the wait modifies the influence of wait duration on aggressive tendencies, such that the information strengthens the influence of wait duration on aggression. Taken together, these findings point to two main conclusions: (1) longer waits can stimulate aggressive tendencies–both directly, because the wait acts as a hindrance stressor, and indirectly, by reducing perceived procedural justice; and (2) providing explanatory information about the wait is useful in reducing aggression only for relatively short wait durations. The longer the wait, the more likely that explanatory information will backfire and increase aggressive tendencies. We explain this reverse effect of providing information by suggesting that the information increases the salience of the waiting situation, which, in turn, primes feelings of procedural injustice. Thus, whereas people waiting who are not provided with information about their wait may be more patient in waiting for their turn, providing such information may increase aggression.

However, our findings in Study 1 could reflect the classic Hawthorne effect [81, 82], also known as the novelty effect, in which an organizational intervention may produce effects in and of itself, independent of the nature of the intervention. To rule out this alternative explanation of our findings, we conducted a second study. Study 2 built on the fact that the ED continued to present the information we initiated after Study 1 was completed. Following Hagger and Luszczynska [70], to verify that the effects we report derived from the information provided and not the Hawthorne effect, we replicated the data collection process a year after Study 1 had ended, using the same tools and measures used in Study 1.

## Study 2

Data were collected one year after Study 1 had ended, in the same ED of the same large hospital. At the time of Study 2, the information provided in Study 1 was still being provided to people waiting in the queue. In Study 2, a research assistant replicated the data collection processes reported in Study 1, using the same surveys.

### Methods

Data were collected over 30 days from 99 care receivers waiting to be served in the ED (M_age_ = 37.18 years, SD = 16.63 years; 49.5% males). Only one research condition–identical to the information condition that was used in Study 1 –was studied. All measures were identical to those used in Study 1. The study received hospital ethics committee approval.

### Results

#### Wait duration, justice perceptions, and aggressive tendencies

The regression analyses confirmed a marginally significant positive influence of wait duration on aggressive tendencies (β = 0.55, p = 0.09), partially supporting Hypothesis 1. This effect was stronger than that found in Study 1 (β = 0.22), and the effect size was substantial (ɳ^2^ = 0.65), indicating that the marginal significance is due to the smaller sample size (n = 155 in the information condition in Study 1, versus n = 99 in Study 2). A t-test comparing the beta values in the two studies showed that the beta value in Study 2 was significantly higher than that in Study 1 (t-difference_(421)_ = 7.24; p < 0.01).

As in Study 1, wait duration was negatively related to perceptions of procedural justice (β = -0.09, p < 0.05), supporting Hypothesis 2, and procedural justice was negatively related to aggression (β = -0.42, p < 0.01), supporting Hypothesis 3. The mediating role of procedural justice in the relationship between wait duration and aggressive tendencies was tested according to Hayes’s [80] model 4, bootstrapped sample = 5000. As in Study 1, wait duration had a negative effect on procedural justice (β = -0.2, p < 0.01, CI = -0.30, -0.10). The indirect effect of wait duration on aggression via procedural justice was significant (β indirect effect = 0.05, CI = 0.01, 0.10; R^2^ = 0.13, p < 0.01, boot = 5000). Thus, the results fully support Hypothesis 4.

Since Study 2 had only one condition (with information), Hypotheses 5 and 6 could not be reexamined. Therefore, to evaluate the long-term effects of providing information, we used a t-test to compare the level of aggressive tendencies in Study 2 with the results obtained in the information condition of Study 1. No significant differences were found between the levels of aggressive tendencies in the information condition of Study 1 (n = 155, M = 1.81, SD = 1.90) and in Study 2 (n = 99, M = 1.84, SD = 1.3) (t (252) = 0.14; p = 0.89).

### Discussion

Study 2 reaffirms Hypotheses 1–4 of Study 1, and rules out the Hawthorne effect. A year after information was initially provided to ED care receivers, the effects of providing information are still apparent. Specifically, aggressive tendencies were lower when the wait was shorter (compared to longer) than the expectation generated by the information.

## General discussion and conclusions

In this research we seek to narrow gaps in the literature regarding the underlying mechanisms behind aggressive tendencies of customers toward employees [8], and in particular, aggressive tendencies of care receivers toward hospital staff. In addition, we suggest and test means of buffering such aggression [9]. Our findings are consistent with existing theories on aggressive tendencies and procedural justice [10, 36, 37, 39–43], and they extend these theories by identifying relevant boundary conditions. First, we resolve the contradiction between existing theories that highlight the bright and dark sides of providing information, whereby information theoretically should reduce aggression by helping people understand the reasons for a wait, but should increase aggression by raising expectations that may not be met. Our findings help delineate the boundaries of the positive effect of explanatory information by showing that such information reduces aggressive tendencies only over short wait durations. In contrast, as a wait lengthens, failure to meet expectations established by explanatory information [83] can lead to feelings of injustice and, in turn, higher levels of aggressive tendencies, as compared with situations in which no information is provided. Finally, we add to existing research on the effects of providing post-hoc information [25] by elucidating the effects of information on people’s reaction to future or current events.

### Limitations and future directions

Our focus on the ED waiting room offers insight into a widespread problem of recurring aggressive tendencies against healthcare professionals. But it also limits the generalizability of our findings to this highly stressful, potentially life-threatening context [82]. The severity of the situation or outcome in this case may influence the effectiveness of the provided information [13, 33]. Therefore, additional research is essential for testing our hypotheses in less-extreme situations (e.g., queues in call centers or banks).

In addition, our study focused on information about organizational procedures, and particularly about wait durations. Future research is needed to unravel the effects of other types of information (beyond wait times) on people’s sense of procedural justice and or aggressive tendencies. Notwithstanding, we show that procedural justice is a mechanism that explains the effects of wait duration on aggressive tendencies, and also the interactive effect of wait duration and provision of information on aggressive tendencies.

That said, we could not, within the scope of this paper, capture the explanatory mechanism underlying the influence of information on the relationship between wait duration and aggressive tendencies. We rely on previous research (e.g., [83], which posits expectations as this mechanism. However, we could not explicitly ask people about their expectations regarding the wait duration, as doing so would prime thoughts about expectations and bias our findings. To empirically examine this theory, future studies should aim to include a measure of the expectations generated by provided information. Moreover, as shown by Shepperd, Sweeny, and Cherry [83], service organizations, such as restaurants, often seek to improve customer satisfaction by creating expectations for wait durations that are longer than the actual expected wait. However, hospitals are obliged to provide care receivers with realistic information about wait durations. Future research should examine whether and how hospital EDs can implement techniques for manipulating expectations in that setting.

A separate concern is that besides generating expectations about wait durations, the provision of information such as that described here could also convey other messages to care receivers–for instance, that the hospital cares for its care receivers, or that the hospital is a well-run organization. Such information could also help people feel less confused or less uncertain about their likely experience in the hospital. Our study design (including the manipulation check) was not sufficient to test these alternative explanations for our findings. This is clearly a limitation of our work, and warrants additional research to rule out the potential confounds.

Finally, our research examined people’s tendency to act with aggression, which we view as a proxy for aggression, building on research [16] showing that aggressive tendencies predict actual aggressive behavior. Clearly, people who report a tendency to act aggressively do not necessarily exercise aggression. Notwithstanding, the effects we observe about the presence of aggressive tendencies may be helpful in thwarting actual instances of aggression. Future research might find ways to examine our theory with actual aggressive behaviors.

### Conclusions

Theoretically, the present research resolves a contradiction between theories that predict that explanatory information should reduce vs. enhance negative outcomes such as aggressive tendencies in the context of waiting. We show that providing information may effectively reduce aggressive tendencies–but only for wait durations that meet the expectations generated by the information. In practical terms, our findings suggest that management–at least in hospital EDs–should analyze customer service situations to ensure that the provided information engenders realistic expectations. When information is effective, its positive effects are robust and continue long after the information is initially introduced.

## Supporting information

S1 Appendix Information provided in the emergency department. (DOCX)

S2 Appendix Study 1 comparison of control variables between the participants who received information and those who did not. (DOCX)
