# Supplementary material for: The effect of waiting on aggressive tendencies toward emergency department staff: Providing information can help but may also backfire
Source: PLoS One. 2020 Jan 29;15(1):e0227729. doi: 10.1371/journal.pone.0227729 (PMC6988907; doi:10.1371/journal.pone.0227729)
Supplement: S1 Appendix — (DOCX) [file pone.0227729.s001.docx]

##### S1 Appendix.

##### Information provided in the Emergency Department.

**The process of patient treatment in the Emergency Department**

**Non-ambulatory ward**

This ward accepts patients who cannot walk. It includes:

Unit A: Internal

Unit B: Trauma, surgical, orthopedics

Unit C: Waiting for hospital admission; intensive-care unit

**Ambulatory ward**

This ward accepts patients who can walk, with internal, surgical, orthopedic or gynecological issues

~170 patients treated daily. Average wait duration - 8 hours.


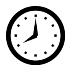

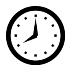


Evaluation and decision-making

**Frequent examinations in the ED:**

Initial nurse examination and disability anamnesis

Physician examination

Blood tests (~two hours for results)

Imaging tests: X-ray, CT, US (~two hours for results)

Consultation with hospital specialists

(waiting duration depends on specialist’s availability)

~130 patients treated daily. Average wait duration - 5 hours.

**Hospitalization**

Waiting durations vary according to the complexity of condition and available space in designated wards.

If designated wards are full, patients will be treated in the Emergency Department until transferred.

**Release**

You will receive a release letter.

Present release letter to the Emergency Department physician promptly for treatment and follow-up recommendations.

Return to reception desk for administrative release.

In subsequent visits, bring physician referral and financial obligation agreement.
