# Supplementary material for: The effect of waiting on aggressive tendencies toward emergency department staff: Providing information can help but may also backfire
Source: PLoS One. 2020 Jan 29;15(1):e0227729. doi: 10.1371/journal.pone.0227729 (PMC6988907; doi:10.1371/journal.pone.0227729)
Supplement: S2 Appendix — (DOCX) [file pone.0227729.s002.docx]

**S2 Appendix.**

**Study 1 comparison of control variables between the participants who received information and those who did not.**

| **Control variable** | **Condition** | **Mean** | **SD.** | **t** | **df** | **Sig. (2-tailed)** | |
| --- | --- | --- | --- | --- | --- | --- | --- |
| **Gender** | **No Information** | .42 | .49 | -1.11 | 326.00 | .27 |  |
|  | **Information** | .48 | .50 |  |  |  |  |
| **Age** | **No Information** | 36.67 | 15.55 | -.02 | 307.00 | .99 |  |
|  | **Information** | 36.70 | 16.51 |  |  |  |  |
| **Years of education** | **No Information** | 12.62 | 4.20 | 1.35 | 326.00 | .18 |  |
|  | **Information** | 11.95 | 4.74 |  |  |  |  |
| **NMI** | **No Information** | 2.47 | 2.31 | -1.32 | 326.00 | .19 |  |
|  | **Information** | 2.80 | 2.16 |  |  |  |  |
| **Time of day** | **No Information** | 1.36 | .10 | -.84 | 313 | .40 |  |
|  | **Information** | 1.17 | .10 |  |  |  |  |

^a^ Measured in hours, such that 1.36 stands for 1:22 pm.
